# Study of the Interaction between Executive Function and Adaptive Behavior at School in Girls with Fragile X Syndrome

**DOI:** 10.3390/genes12081108

**Published:** 2021-07-21

**Authors:** Lorena Joga-Elvira, Jennifer Martinez-Olmo, María-Luisa Joga, Carlos Jacas, Ana Roche-Martínez, Carme Brun-Gasca

**Affiliations:** 1 Consorcio Corporación Sanitaria Parc Tauli, 08208 Sabadell, Spain; j.m.olmo@hotmail.com (J.M.-O.); ana.roche.mar@gmail.com (A.R.-M.); carme.brun@uab.cat (C.B.-G.); 2 Departamento de Psiquiatría y Medicina Legal, Universitat Autònoma de Barcelona, Bellaterra, 08193 Barcelona, Spain; 3 Hospital San Joan de Deu, 08035 Barcelona, Spain; mjoga@sjdhospitalbarcelona.org; 4 Servicio de Psiquiatría, Hospital Universitari Vall d’Hebron, 08035 Barcelona, Spain; cjacas1@gmail.com; 5 Departament de Psicología Clínica i de la Salut, Universitat Autònoma de Barcelona, Bellaterra, 08193 Barcelona, Spain

**Keywords:** fragile X syndrome, young females, executive function, adaptive behavior, school

## Abstract

The aim of this research is to analyze the relationship between executive functions and adaptive behavior in girls with Fragile X syndrome (FXS) in the school setting. This study is part of a larger investigation conducted at the Hospital Parc Tauli in Sabadell. The sample consists of a total of 40 girls (26 with FXS and 14 control) aged 7–16 years, who were administered different neuropsychological tests (WISC-V, NEPSY-II, WCST, TOL) and questionnaires answered by teachers (ABAS-II, BRIEF 2, ADHD Rating Scale). The results show that there is a greater interaction between some areas of executive function (cognitive flexibility, auditory attention, and visual abstraction capacity) and certain areas of adaptive behavior (conceptual, practical, social, and total domains) in the FXS group than in the control group. These results suggest that an alteration in the executive functions was affecting the daily functioning of the girls with FXS to a greater extent.

## 1. Introduction

Fragile X syndrome (FXS) is the most common cause of sex-linked inherited intellectual disability. It is a genetic condition that occurs as a consequence of amplification of the CGG triplet of the FMR1 (fragile X mental retardation 1) gene located on Xq27.3 [1,2]. This mutation leads to a total absence or partial reduction of FMRP protein levels, which is essential for neurodevelopment. There is a classification of individuals according to the number of CGG triplet repeats, summarized in Table 1 [3].

The clinical phenotype usually presents more severely in males than in females, since in females the unaffected X chromosome can synthesize some amount of FMRP [4]. This fact has meant that most of the previous scientific literature has focused on studying the clinical phenotype of males.

In the case of females affected by FXS, we found a great variability in their neurocognitive phenotype. Their intellectual ability can range from intellectual disability (mild–moderate) to within the average [1]. A total of 30% of girls shows symptoms of attention deficit hyperactivity disorder (ADHD), and a very distinctive feature is social anxiety. Other behavioral characteristics include stereotypes (such as fluttering), extreme shyness, perseveration, emotional lability, aggressiveness, and pragmatic speech difficulties [1].

In much of the research carried out to date, executive function (EF) is described as a domain of cognitive weakness in girls with FXS. EF is a construct that encompasses a set of skills needed to carry out a goal directed behavior. Attention, inhibitory control, working memory, cognitive flexibility, processing speed, and planning are included [5]. Attention is understood as the ability to initiate, maintain, or shift attentional focus selectively or divided among different tasks [6]. Inhibitory control can be described as the ability to suppress an automatic response to a stimulus [6]. Working memory allows us to maintain and process information mentally. Cognitive flexibility is the ability to switch from one mental schema to another [6]. Processing speed is people’s ability to process a stimulus and respond to it [5]. Last, there is planning, which is involved in problem solving and is responsible for efficiently organizing information to perform a task successfully [6].

In a systematic review it was found that girls with FXS presented worse performance in working memory tasks, inhibitory control, and processing speed that the control group (CG) [5]. Another investigation showed difficulties in information processing, planning, maintaining attitude, abstract concept formation, and perseverative thinking [2].

In another study of 50 women aged between 18 and 58 years (16 negative FXS, 12 with FXS full mutation, and 22 with premutation) it was found that women with FXS obtained significantly lower IQ scores. The results obtained also suggest a deficit in verbal working memory in the group of women with FXS [7]. Along similar lines, in an extensive review it was found that perseverative errors are the most consistent alteration in the profile of women with FXS [5].

Adaptive behavior is defined as the set of conceptual, social, and practical skills that people learn to be able to function in daily life. In other words, it is the degree or effectiveness with which people meet the requirements of independence and social responsibility expected for their age and cultural group [8]. In females with FXS, a degradation of their adaptive behavior as they grow older and the environment becomes increasingly demanding has been described [9]. In another longitudinal study, adaptive behavior in 275 boys and girls with FXS compared to 225 control children aged 2–18 years was analyzed. The study concluded that as the children with FXS grew older, they scored worse on adaptive behavior in both groups. In the case of girls, the difficulties found were in the area of communication. The authors concluded that it was important to take this decline in adaptive behavior into account and make it part of intervention programs [10].

Only two studies were found that focused on the influence of EF on independent living skills. The first studied the role of EF in independent living skills in adolescents and young adults with FXS (*n* = 18) and Turner syndrome (*n* = 16). No significant differences were found between the two groups, and it was concluded that the poorer performance in the tasks used to assess EF was attributable to their IQ and not to the fact of presenting the FXS condition. Despite there being no significant differences between the groups, it was concluded that EF (and specifically phonemic verbal fluency) are related to basic daily living skills [6]. In the second study, the aim was to describe the relationship between EF and level of performance in the following areas of the neurocognitive profile: linguistic functions, quantitative reasoning, social perception, social skills profile, and adaptive behavior. It was found that better verbal abstraction ability has a positive impact on the conceptual adaptation domain; better inhibition, self-monitoring, emotional control, and working memory skills have a positive impact on the degree of social adaptation and self-direction ability; girls with better organizational skills (planning/organization and organization of materials) have a higher ability in self-direction; a higher level of emotional and behavioral regulation leads to better social adaptation; and higher cognitive flexibility improves self-direction, social adaptation, and conceptual adaptation ability [11]. It should be noted that in this research the information analyzed focused on the family setting.

Thus, the main objective of the present study was to analyze the relationship between executive functions and adaptive behavior in girls with FXS in the school setting. To this effect, and based on the previous literature, the following hypothesis was proposed: better performance in some EF tasks implies better scores in some aspects of adaptive behavior. This relationship will occur differently with the presence of an FXS diagnosis.

## 2. Materials and Methods

The current research is the continuation of a previous investigation in which the relationship between linguistic functions and social perception, quantitative reasoning, and adaptive behavior was investigated. It was approved by the Parc Tauli Hospital’s Ethics Committee (reference 2016595). The Parc Tauli Hospital, the Spanish FXS Federation, different associations of patients with FXS (Catalonia, Basque Country, Madrid, and Valencia), and patients with genetic diseases (D’Genes Murcia) collaborated in the dissemination of the project to facilitate the recruitment of participants. Likewise, a call was made via professional social networks such as Linked-In and emails sent to the psycho-pedagogical advisory teams in the area and to the special education colleges.

Informed consent was obtained from the legal guardians of all the participants. The tests to be performed on the young girls and adolescents were also explained. If they had refused to perform the tests, the evaluation would not have been carried out. All of them verbally agreed to perform the tests.

### 2.1. Participants

A total of 40 girls aged between 7 and 16 years took part in the study. They were divided into the following two groups:

- FXS group: *n* = 26. Participants recruited through the Parc Tauli Hospital and the different patient associations.
- Control group (CG): *n* = 14. Participants were recruited through outpatients’ appointments at the Neuropediatric Unit of Sabadell’s Parc Tauli Hospital and standard and special education schools in the area.

The inclusion and exclusion criteria were as follows:

#### 2.1.1. Inclusion Criteria

- FXS group: female, aged between 7 and 16 years, FXS confirmed by genetic study (>200 repetitions).
- Control Group: female, aged between 7 and 16 years.

#### 2.1.2. Exclusion Criteria

- FXS criteria: presence of comorbid autism spectrum disorder (ASD) or ADHD, absence of expressive language, acquired neurological disorders (epilepsy, head injury).
- Control Group: meeting the diagnostic criteria for ASD or ADHD, absence of expressive language, acquired neurological disorders (epilepsy, head injury) and other cognitive-behavioral disorders. Participants without ID did not undergo a genetic study for ethical reasons, but the subjects were questioned explicitly about their family history of FXS and the presence of clinical symptoms compatible with an FXS diagnosis.

In both groups, participants who met all the DSM-5 criteria for the diagnosis of ASD and ADHD were excluded from the sample. Participants who only showed some symptoms were not excluded. The assessment was performed by an expert in pediatric neurology, psychology, or psychiatry.

### 2.2. Measures and Procedure

The assessment sessions for the participants from the Barcelona Metropolitan Area took place in Sabadell’s Parc Tauli Hospital. The lead investigator travelled to the autonomous regions where the rest of the participants lived to carry out the assessments, thus ensuring as little inconvenience as possible for the participants and their families. The participating autonomous communities were Catalonia, Madrid, the Basque Country, Galicia, Valencia, and Murcia.

First, a document explaining the project was produced and sent to the patient associations for dissemination among the families. When a family showed interest in taking part in the study, the lead investigator would travel to the pertinent autonomous region on previously agreed days to carry out the assessments in the facilities provided by the associations. The assessment was divided into three separate one-hour sessions to ensure that participants did not become overtired. Last, teachers were asked to complete the corresponding questionnaires. Due to the general and reading comprehension difficulties shown by most of the subjects, the administration of self-reported questionnaires was not possible.

### 2.3. Measures and Scales

The Wechsler Intelligence Scale for Children-V was used to assess General Cognitive Ability [12].

The following aspects of executive function were used as independent variables: Visual Working Memory (WISC-V. Picture Span subtest), Auditory Working Memory (WISC-V. Digits and Letters and Numbers subtest) [12]; Auditory Attention (NEPSY-II. Auditory Attention subtest), Cognitive Flexibility (NEPSY-II. Cognitive Flexibility subtest, total correct), Inhibition (NEPSY-II. Inhibition subtest, total correct), [13]; Cognitive Flexibility (Wisconsin Card Storing Test. Learning to learn) [14]; Problem Solving (Tower of London. Total Correct), Problem Solving Efficiency (Tower of London. Total Move), and Breaking Rules (Tower of London. Total Rule Violation) [15]. The behavioral assessment of executive function-2 (BRIEF-2), school version, was used as an ecological measure of executive function [16].

The following questionnaires and scales were used to assess the behavioral variables: ADHD Rating Scale (teachers’ version) to assess the ADHD Symptomatology [17] and the Adaptive Behavior Assessment System—Second Edition Spanish version (ABAS-II), teachers’ version, as a measure of Adaptive Behavior [8].

### 2.4. Genetic Study

At Hospital Parc Tauli, genetic studies are performed using the Asuragen commercial kit, which includes two PCRs. The AmplideX^®^ PCR/CE FMR1 kit is an in vitro diagnostic tool designed for professional use in clinical laboratories to amplify and detect the cytosine-guanine-guanine triplet (CGG) repeat sequences in the 5′ untranslated regions of the Fragile X mental retardation gene 1 (FMR1). The kit is intended as an aid in diagnosing Fragile X syndrome and associated disorders such as tremor/ataxia syndrome (FX-TAS), and primary ovarian insufficiency (FX-POI) through determination of the number of CGG repeats up to 200 CGGs and detection of alleles with more than 200 CGGs. The test consists of a polymerase chain reaction (PCR) of genomic DNA purified from whole blood or buccal cells, followed by fragment measurement on a general laboratory-validated genetic analyzer or capillary electrophoresis platform, and conversion of product size to the number of CGG repeats. The kit includes reagents to perform 2 types of PCRs: gene-specific PCR (GS-PCR) and PCR with CGG repeat primers. Gene-specific PCR uses two primers that span the CGG repeat region. The PCR products from the gene-specific primers represent full-length alleles. PCR with CGG repeat primers differs from the more conventional gene-specific PCR by the addition of a third primer, which is complementary to the triplet repeat region of the FMR1 gene. The resulting electropherogram includes the full-length PCR products generated from the gene-specific primers spanning the CGG repeat region and the CGG repeat peaks obtained by RP PCR. The full-length gene-specific peaks are similar in the two methods. The PCR products with CGG repeat primers correspond to the individual PCR amplicons of each combination of the repeat primer with the reverse primer. The peaks corresponding to the CGG repeats are separated by 3 bp, as expected. The peak profile provides confirmatory information about a sample, including zygosity and the presence of interspersed AGG sequences.

### 2.5. Statistical Analysis

The statistical analysis was carried out using the SPSS Statistics for Windows Version 25.0 software.

Student′s t-test was used for the demographic data and the control variables (IQ, age, socioeconomic level, and ADHD symptoms). For the binary variables, an interaction model was made by creating contingency tables between the tests performed with the patients and the questionnaires filled out by the teachers, comparing the group of girls with FXS and the control group with a 95% confidence interval, and the variables were also selected by adjusted r^2^ > 0.60.

## 3. Results

Regarding IQ, 50% of the participants in the FXS group in our sample scored in the range of mild/moderate ID, while the remaining 50% scored in the medium/medium-low range. No significant differences were found in the control variables age, IQ, ADHD symptoms, and socioeconomic status. (Table 1).

Once the data were analyzed, statistically significant differences were found between the two groups, with the FXS group presenting a stronger relationship between certain EF and some adaptive behavior skills.

A relationship was found between the following variables: visual abstraction ability and conceptual, practical, social, and total adaptive behavior domains. Correct auditory attention and social and practical domains. Auditory attention commission errors and conceptual, social, practical, and total adaptive behavior domains. Auditory attention omission errors and social and practical domains. Auditory attention inhibition errors and practical domain. Correct cognitive flexibility and social and practical domains. Cognitive flexibility errors of commission and conceptual, social, practical, and total adaptive behavior domains. Cognitive flexibility omission errors and total adaptive behavior domain. Cognitive flexibility inhibition errors and social and practical domains. Global index of executive function and conceptual), social, practical, and total adaptive behavior domains (Table 2).

## 4. Discussion

The aim of the present research was to study the relationship between EF and performance in adaptive behavior at school. Having analyzed the results, we can conclude that cognitive flexibility and auditory attention are two areas of EF that have a greater influence on adaptive behavior in girls with FXS than on the CG.

Both variables were obtained from the auditory attention and cognitive flexibility subtest of the NEPSY-II battery. Focusing on the errors of commission of both tests, the authors point out that these may be due to impulsivity, inattention, or slow response [13]. Considering that there were no significant differences in ADHD symptoms between the two groups, as mentioned above, attention deficit and impulsivity were ruled out as probable causes. Therefore, a possible explanation would be that the girls with FXS have a significantly slower reaction time (greater response latency) than the girls in the control group. The fact of having a longer reaction time has a negative impact on the conceptual and practical domains of their adaptive behavior because having an adequate response time is a very important factor to be able to adapt adequately in the conceptual (e.g., being able to follow the teachers′ explanations, finish exams on time, etc.) and practical (e.g., crossing the street when the traffic light turns green) domains. These results are consistent with those obtained in a previous investigation in which it was concluded that the girls with FXS were substantially slower in tasks requiring fast reaction time [18].

Focusing on the omission errors, in this case the relationship is with the social, practical, and total adaptive behavior domains in the case of cognitive flexibility, and with the social and practical domains in the case of auditory attention. Poor performance in this test may be due to low vigilance [13]. Being able to pay attention in a sustained manner and without omitting any stimuli is fundamental for both social relations and for being able to develop autonomously in the environment. The same goes for the ability to adapt flexibly to changes in the environment. Omitting and not perceiving all stimuli has a significant impact on the ability to modify behavior according to the changing demands of the environment.

Next, we discuss the inhibition errors, which affect the social and practical domains in the case of cognitive flexibility and only the practical domain in the case of auditory attention. These errors may be due to impulsivity or difficulty in switching from a previously learned behavior to a new response [13]. A better capacity for inhibition has a positive impact on the degree of social and practice adaptation. The ability to suppress an automatic response and environmental distractions are skills that help us adapt to the demands of the environment. Consequently, the more able a subject is to adapt to these demands, the greater the level of adaptation they will achieve. These results agree with the findings of Joga et al. (2021), where it was found that a better capacity for inhibition has a positive impact on the degree of social adaptation and the capacity for self-direction [11].

Regarding the correct answers, the relationship was found with the social and practical domains of adaptive behavior in the cases of both cognitive behavior and auditory attention. Cognitive flexibility is very important in any social relationship to be able to adapt to turns and changes of conversation, game rules, etc. Auditory attention is also essential since the auditory channel is fundamental for oral communication. The practical domain is the one that would encompass more basic activities of daily life such as self-care and handling money to go shopping. To carry out these types of activities effectively, it is essential to be able to communicate with the people around us (for example, to ask for food from the salesclerk) and to know how to change from one activity to another during the day.

Furthermore, a relationship was found between visual abstraction capacity and all domains of adaptive behavior (conceptual, social, practical, and total). These results are in line with those found by Joga et al. (2021), where it was found that a better verbal abstraction ability has a positive impact on the domain of conceptual adaptation [11]. To this effect, we can conclude that a better ability to process stimuli abstractly has a positive impact on the adaptive behavior of girls with FXS.

Last, a relationship was found between global executive functioning (global index of executive function) and all domains of adaptive behavior (conceptual, social, practical, and total), which indicates that having executive dysfunction had a greater impact on all areas of adaptive behavior in the girls of the FXS group.

### Strengths and Limitations

One of the limitations of this study is the relatively small size of the sample. However, despite only 26 girls with FXS participating in the study, it is still the largest sample of girls of child-juvenile age collected to date in Spain, and one of the largest at an international level.

Another limitation was the great difficulty we had in finding many girls for the CG. Nonetheless, there were no significant differences between the groups in terms of the control variables, age, IQ, ADHD symptoms, and socioeconomical status.

Furthermore, while the girls with ID in the control group had undergone a genetic analysis ruling out FXS, at no time did the girls without ID undergo a genetic study for ethical reasons. However, the participants were evaluated clinically to assess whether they presented symptoms compatible with FXS (physical traits, cognitive-behavioral characteristics), and they were asked specifically about any previous family history of FXS.

## 5. Conclusions

The results show that there is a greater interaction between some areas of executive function (cognitive flexibility, auditory attention, and visual abstraction capacity) and certain areas of adaptive behavior (conceptual, practical, social, and total domains) in the FXS group than in the control group. These results suggest that an alteration in the executive functions was affecting the daily functioning of the girls with FXS to a greater extent.

## Figures and Tables

**Table 1:** Descriptive measures and distribution of age, IQ, ADHD Symptoms, and socioeconomical status.

Variables	FXS Group *n* = 26	Control Group *n* = 14	Significance
Age M (SD)	10.58 (3.384)	10.50 (2.345)	0.940
I QM (SD)	71.50 (14.795)	76.36 (16.80)	0.351
ADHD symptoms M (SD)	81.54 (4.341)	68.29 (6.984)	0.104
Socioeconomical status			0.211
Low	3.8%	16.7%	
Medium	88.5%	75%	
High	7.7%	8.3%	

FXS = Fragile X Syndrome. IQ = intelligence quotient. ADHD = attention deficit hyperactivity disorder. M = mean. SD = standard deviation. M = mean.

**Table 2:** Results of interaction between executive functions and adaptive behavior.

Variables	Conceptual Domain	Social Domain	Practical Domain	Total Adaptive Behavior
Cognitive flexibility comission errors	*p* < 0.001 r^2^ = 0.607	*p* < 0.001 r^2^ = 0.666	*p* < 0.001 r^2^ = 0.783	*p* < 0.001 r^2^ = 0.670
Cognitive flexibility omission errors	*p* < 0.001 r^2^ = 0.579	*p* < 0.001 r^2^ = 0.724	*p* < 0.001 r^2^ = 0.718	*p* < 0.001 r^2^ = 0.602
Cognitive flexibility inhibition errors	*p* < 0.001 r^2^ = 0.579	*p* < 0.001 r^2^ = 0.614	*p* < 0.001 r^2^ = 0.716	*p* < 0.001 r^2^ = 0.584
Cognitive flexibility correct	*p* < 0.001 r^2^ = 0.525	*p* < 0.001 r^2^ = 0.647	*p* < 0.001 r^2^ = 0.723	*p* < 0.001 r^2^ = 0.578
Auditive attention comission errors	*p* < 0.001 r^2^ = 0.603	*p* < 0.001 r^2^ = 0.646	*p* < 0.001 r^2^ = 0.742	*p* < 0.001 r^2^ = 0.624
Auditive attention omission errors	*p* < 0.001 r^2^ = 0.501	*p* < 0.001 r^2^ = 0.616	*p* < 0.001 r^2^ = 0.685	*p* < 0.001 r^2^ = 0.516
Auditive attention inhibition errors	*p* < 0.001 r^2^ = 0.515	*p* < 0.001 r^2^ = 0.588	*p* < 0.001 r^2^ = 0.691	*p* < 0.001 r^2^ = 0.537
Auditive attention correct	*p* < 0.001 r^2^ = 0.492	*p* < 0.001 r^2^ = 0.589	*p* < 0.001 r^2^ = 0.685	*p* < 0.001 r^2^ = 0.508
Visual abstraction	*p* < 0.001 r^2^ = 0.644	*p* < 0.001 r^2^ = 0.627	*p* < 0.001 r^2^ = 0.787	*p* < 0.001 r^2^ = 0.659
Overall function index Executive Function	*p* < 0.001 r^2^ = 0.728	*p* < 0.001 r^2^ = 0.738	*p* < 0.001 r^2^ = 0.802	*p* < 0.001 r^2^ = 0.744

## Data Availability

Data available under request.
